# Resolvins Decrease Oxidative Stress Mediated Macrophage and Epithelial Cell Interaction through Decreased Cytokine Secretion

**DOI:** 10.1371/journal.pone.0136755

**Published:** 2015-08-28

**Authors:** Ruan Cox, Oluwakemi Phillips, Jutaro Fukumoto, Itsuko Fukumoto, Prasanna Tamarapu Parthasarathy, Maria Mandry, Young Cho, Richard Lockey, Narasaiah Kolliputi

**Affiliations:** 1 Department of Internal Medicine, Division of Allergy and Immunology, Morsani College of Medicine, University of South Florida, Tampa, Florida, United States of America; 2 Department of Molecular Medicine, Morsani College of Medicine, University of South Florida, Tampa, Florida, United States of America; Emory University School of Medicine, UNITED STATES

## Abstract

**Background:**

Inflammation is a key hallmark of ALI and is mediated through ungoverned cytokine signaling. One such cytokine, interleukin-1beta (IL-1β) has been demonstrated to be the most bioactive cytokine in ALI patients. Macrophages are the key players responsible for IL-1β secretion into the alveolar space. Following the binding of IL-1β to its receptor, “activated” alveolar epithelial cells show enhanced barrier dysfunction, adhesion molecule expression, cytokine secretion, and leukocyte attachment. More importantly, it is an important communication molecule between the macrophage and alveolar epithelium. While the molecular determinants of this inflammatory event have been well documented, endogenous resolution processes that decrease IL-1β secretion and resolve alveolar epithelial cell activation and tissue inflammation have not been well characterized. Lipid mediator Aspirin-Triggered Resolvin D1 (AT-RvD1) has demonstrated potent pro-resolutionary effects *in vivo* models of lung injury; however, the contribution of the alveoli to the protective benefits of this molecule has not been well documented. In this study, we demonstrate that AT-RvD1 treatment lead to a significant decrease in oxidant induced macrophage IL-1β secretion and production, IL-1β-mediated cytokine secretion, adhesion molecule expression, leukocyte adhesion and inflammatory signaling.

**Methods:**

THP-1 macrophages were treated with hydrogen peroxide and extracellular ATP in the presence or absence of AT-RvD1 (1000–0.1 nM). A549 alveolar-like epithelial cells were treated with IL-1β (10 ng/mL) in the presence or absence of AT-RvD1 (0.1 μM). Following treatment, cell lysate and cell culture supernatants were collected for Western blot, qPCR and ELISA analysis of pro-inflammatory molecules. Functional consequences of IL-1β induced alveolar epithelial cell and macrophage activation were also measured following treatment with IL-1β ± AT-RvD1.

**Results:**

Results demonstrate that macrophages exposed to H_2_O_2_ and ATP in the presence of resolvins show decreased IL-1β production and activity. A549 cells treated with IL-1β in the presence of AT-RvD1 show a reduced level of proinflammatory cytokines IL-6 and IL-8. Further, IL-1β-mediated adhesion molecule expression was also reduced with AT-RvD1 treatment, which was correlated with decreased leukocyte adhesion. AT-RvD1 treatment demonstrated reduced MAP-Kinase signaling. Taken together, our results demonstrate AT-RvD1 treatment reduced IL-1β-mediated alveolar epithelial cell activation. This is a key step in unraveling the protective effects of resolvins, especially AT-RvD1, during injury.

## Introduction

Airway inflammation is a key hallmark in inflammatory lung disease such as acute lung injury (ALI) and acute respiratory distress syndrome (ARDS) [1, 2]. Following injury there is an immediate release of proinflammatory mediators that serve to enhance the inflammatory response. One of these inflammatory mediators, IL-1β is the most bioactive cytokine in the lungs of ALI patients [3]. IL-1β when secreted into the alveolar space (mainly by alveolar macrophages), can act through its receptor IL-1R to upregulate mechanisms associated with vascular and tissue remodeling, cytokine and chemokine expression, cellular attachment, as well would repair [4–6]. *In vitro* murine models have shown that proinflammatory mediators play a key role in the pathology of ALI [7–16]. IL-1β’s cellular binding partners include macrophages, endothelial cells, and the alveolar epithelium. The alveolar epithelium is a key regulator of the proinflammatory and anti-inflammatory immune response.

The role of alveolar epithelium in inflammation is still not yet clear. Previously thought to participate in surfactant production and barrier function, more studies are elucidating the role of the alveolar epithelium in the inflammatory stage in response to proinflammatory mediators such as IL-1β [17]. IL-1β serves to activate the alveolar epithelium and enhances the expression of proinflammatory products such as cytokines, chemokines, adhesion molecules, and pro- inflammatory lipid mediators [3]. IL-1β binding has also been demonstrated to greatly diminish the physical barrier properties and permeability of the alveolar epithelium through decreased tight junction formation. More important to the persistent inflammation seen in ALI, the alveolar epithelium plays an integral part in the recruitment of circulating leukocytes to the area of injury [18, 19]. The release of cytokines and expression of adhesion molecules by the activated alveolar epithelium aids in the cytokine gradient and attachment points used by leukocytes to migrate into the alveolar space. The consistent uncontrolled extravasation of these leukocytes, such as neutrophils and monocytes, into injurious tissue is well documented to be a key event in the development and progression of inflammatory lung disease [20–23]; however, the endogenous resolution mechanisms and catabasic processes used by our body to resolve this imbalance has yet to be clarified.

Resolution phase interaction products (resolvins) are lipid mediators derived from omega-i3 free fatty acids eicosapentaenoic acid (EPA) and docosahexaenoic acid (DHA) [24–27]. In the presence of aspirin, the aspirin-triggered forms of these lipid mediators are produced. Aspirin-triggered resolvins, unlike the traditional resolvins, are resistant to oxidation and therefore are able to show prolonged effect in comparison with traditional resolvins [25]. We have recently demonstrated the ability of aspirin triggered-resolvin D1 to enhance resolution of hyperoxic acute lung injury (HALI) [28]. Resolvins show therapeutic promise in many different disease models [24, 25, 29]; however, their role on the activation of the alveolar epithelium has not been elucidated. In this study, we investigated the effects of aspirin-triggered resolvin D1 (AT-RvD1) on IL-1β-induced alveolar epithelial cell activation. Here, we have chosen H_2_O_2_ to induce oxidant stress in cell culture because it directly confers oxidant injury which is essential feature in the pathogenesis of HALI. Further, H_2_O_2_ is elevated in the expired gas and urine of patients with ARDS [30].

The results indicate that AT-RvD1 modulates IL-1β-mediated epithelial cell cytokine secretion and adhesion molecule expression. Further, our results also demonstrated that AT-RvD1 also inhibit IL-1β-stimulated leukocyte adhesion. In addition, AT-RvD1 decreased the activation of the p38 and ERK map kinase pathways, which are known to be involved in the propagation of the inflammatory cascade in the alveolar epithelium. Our results suggest that targeting the alveolar epithelium with resolvins, is a novel way to reduce inflammation in the alveolar space.

## Materials and Methods

### Cell Culture

A549 cells are a human alveolar epithelial cell line derived from a human lung adenocarcinoma patient. THP1 cells are a human monocytic cell line obtained from an acute myeloid leukemia patient. Both cell lines were purchased from American Type Culture Collection (ATCC, Manassas, VA). A549 cells and THP1 express all the components necessary to serve as an effective cell line to model epithelial cell activation and monocyte adhesion, respectively. A549 cells were cultured in Dulbecco’s Modified Eagle Medium/F12 Nutrient mix (1:1 ratio, Gibco, Grand Island, NY) complete with 10% fetal bovine serum (FBS, Gemini Biologicals, Sacramento, CA) and 1% penicillin/streptomycin mixture (P/S, Gibco, Grand Island, NY). THP1 cells were used for cytokine, caspase and adhesion assays and were cultured in RPMI 1640 medium (Gibco, Grand Island, NY) complete with 10% FBS (Gemini Biologicals, Sacramento, CA), 1% P/S and 1% sodium pyruvate (Gibco, Grand Island, NY). Both cell lines were maintained in a humidified atmosphere at 37°C and 5% concentration. Cells were treated as mentioned in each section.

### Cytokine Analysis

A549 cells were seeded in 12 well plates at a density of 0.5 x 10^6^ cells/mL. Following 24 hr incubation to reach confluence, cells were serum starved for 6 hours. After serum starvation A549 cells were treated with IL-1β (10 ng/mL, R&D Systems, Minneapolis, MN) in the presence or absence of aspirin-triggered resolvin D1 (100 nM, AT-RvD1, Caymen Chemicals, Ann Arbor, MI) for 6 hours. Optimization of AT-RvD1 treatment was done using varying AT-RvD1 concentrations in THP1 experiments (0.1–1000 nM). We did not see a change in cell viability following AT-RvD1 treatment as measured by trypan blue dye exclusion assay (S1 Fig). Cells receiving AT-RvD1 were pretreated with AT-RvD1 for 30 minutes prior to addition of IL-1β or vehicle. THP-1 cells were seeded in 12 well plates at a density of 0.5 x 10^6^ cells per well. They were allowed to reach 85–90% confluence and then were serum starved in the presence of priming agent phorbol-12-myristate-13-acetate (PMA, 10 ng/mL). Following treatment with PMA cells were primed with LPS to generate pro-IL-1β and then treat with inflammasome activators ATP and hydrogen peroxide in the presence or absence of docosahexaenoic acid, EPA, Resolvin D1 (RvD1), Resolvin D2 (RvD2) or AT-RvD1, all at indicated dosages. All resolvins as well as DHA and EPA were obtained from Caymen Chemicals (Ann Arbor, MI). Following the treatment of A549 and THP1 cells, supernatants were collected and used for cytokine analysis via ELISA. ELISA for IL-1β (E-bioscience, San Diego, CA), IL-6 (E-bioscience, San Diego, CA) and IL-8 (R&D Systems, Minneapolis, MN) was performed following the manufacturer’s instructions. Absorbance measurements were made using an absorbance plate reader (Biotek, Winooski, VT) and concentrations were obtained using manufacturer provided standards. ELISA for active caspase-1 (R&D Systems, Minneapolis, MN) in THP1 cells was performed using cell lysates and measured using absorbance readings.

### Quantitative Real Time-PCR (qPCR)

A549 cells were seeded in 12 well plates at a density of 0.5 x 10^6^ cells/mL. Following 24 hr incubation to reach confluence, cells were serum starved for 6 hours. After serum starvation A549 cells were treated with IL-1β (10 ng/mL) in the presence or absence of AT-RvD1 (100 nM) for 6 hours. Following treatment, cells were resuspended in Trizol (Invitrogen, Grand Island, NY) and mRNA was harvested following the manufacturer’s instructions. 1 μg of RNA for each sample was reverse transcribed into cDNA using the iSCRIPT cDNA synthesis kit (Bio-Rad, Hercules, CA) according to the manufacturer’s instructions. Forward and reverse primers for 18S ribosomal RNA as well as ICAM-1 were purchased from Integrated DNA Technologies (Coralville, IA). Primers were used in a final concentration of 100 nM and assays were done in triplicate. Human ICAM-1 and 18S ribosomal DNA were amplified using the following primers: ICAM-1F:5’-AGC AAT GTG CAA GAA GAT AGC CAA-3’, ICAM-1R: 5’-GGT CCC CTG CGT GTT CCA CC-3’, 18sF: 5′-TAA CGA ACG AGA CTC TGG CAT-3′ and 18sR: 5′-CGG ACA TCT AAG GGC ATC ACA G-3′. qPCR was performed using the SYBR green master mix and CFX96 system (Bio-Rad, Hercules, CA). Normalization of ICAM-1 expression was achieved by comparing the expression of 18S ribosomal RNA for the corresponding sample.

### Epithelial Cell-Monocyte Adhesion Assay

The epithelial monocyte assay was performed as previously described with minor modification [31]. Briefly, A549 cells were activated for 6 hours with IL-1β in the presence or absence of AT-RvD1. After treatment, the cells were washed with PBS and new media was added to each well. 30 minutes prior to end of the 6 hr incubation, monocytes were loaded with calcein AM dye (Life Technologies, Carlsbad, CA) and immediately added in the activated A549 cells. After a 30 minute incubation period, unbound monocytes were washed away with PBS twice and fluorescent images were taken.

### Calcein AM loading of monocytes

Calcein AM was purchased from Invitrogen (Grand Island, NY) and diluted to a working stock of 1 mM and stored in -20°C in light sensitive microcentrifuge tubes. Prior to the experiment, THP1 cells were loaded with calcein AM at a final concentration of 10 μM for 30 minutes at normal cell culture conditions in a humidified incubator. Following incubation, cells were washed twice with PBS (without Ca^2+^ and Mg^2+^) and diluted at a final concentration of 0.5 x 10^6^ cells/mL prior to the addition of cells to the plates containing IL-1β activated A549 cells. 0.5 x 10^6^ cells/mL was added to each treatment for microscopy analysis. For plate reader analysis, 5 x 10^4^ THP1 cells were used.

### Adhesion assay for alveolar epithelial cell-monocyte interaction

1 x 10^6^ and 1 x 10^5^ A549 cells were seeded in 12 well and 96 well plates, respectively. After overnight incubation in complete medium, cells were serum starved for 6 hours. After serum starvation A549 cells were treated with IL-1β (10 ng/mL) in the presence or absence of AT-RvD1 (100 nM) for 6 hours. Cells receiving resolvins were pretreated with AT-RvD1 for 30 minutes prior to addition of IL-1β or vehicle. Following treatment, the aforementioned amount of THP1 cells were added to each plate and co-incubated with A549 cells for 30 minutes. After incubation of A549 cells with THP1 cells, the plate washed with PBS four times to ensure only bound THP1 cells were left in the plate. Microscopy (12 well plates) as well as relative fluorescence intensity measurements (96 well plates) were used to analyze monocyte adhesion to treated A549 cells. Images were taken and plates were read using excitation-emission spectra of Ex: 495nm/Em: 515nm.

### Electric Cell-Substrate Impedance Sensing (ECIS)

A549 cells were seeded on 8W10E+ ECIS arrays (Applied Biophysics, Troy, NY) at a density of 0.5 x 10^6^ cells/mL. Cells were grown to a confluence as measured by a reading of 10 μF on the ECIS System (Applied Biophysics, Troy, NY). After achieving confluence, A549 cells were subjected to serum starvation and were treated with IL-1β (10 ng/mL) in the presence or absence of AT-RvD1 (100 nM) for 6 hours. Cells receiving resolvins were pretreated with AT-RvD1 for 30 minutes prior to addition of IL-1β or vehicle. Resistance measurements were taken for a period of 48 hours. Three samples were used and 4 measurements were taken at each time point to mitigate inter- and intra- sample variability. Culture medium served as the electrode and barrier function was analyzed as a function of impedance of a cell-covered electrode. A 1-V, 4,000-Hz alternating current signal was supplied through a 1-MΩ resistor to approximate a constant-current source. ECMS 1.0 software (CET, Coralville, IA) was used to measure the in-phase voltage resistance and capacitance. Epithelial barrier function of A549 cells was expressed as TER, which was normalized to a time-zero baseline. Epithelial monolayers that did not achieve a baseline TER of 5,000 Ω or higher were excluded from this study.

### Western Blot Analysis

As with ELISA, A549 cells were seeded in 12 well plates at a density of 0.5 x 10^6^ cells/mL. Following 24 hr incubation to reach confluence, cells were serum starved for 6 hours. After serum starvation A549 cells were treated with IL-1β (10 ng/mL) in the presence or absence of AT-RvD1 (100 nM) for 6 hours. Cells receiving resolvins were pretreated with AT-RvD1 for 30 minutes prior to addition of IL-1β or vehicle. Following A549 cell treatment, cell lysates were collected using RIPA Buffer (25 mM Tris-HCl (pH 7.6), 150 mM NaCl, 1% NP-40, 1% sodium deoxycholate, 0.1% SDS, Thermo Scientific Pierce, Rockford, IL). After lysate extraction, protein concentration was determined with a BCA assay (Thermo Scientific Pierce, Rockford, IL) following the manufacturer’s specific instructions. 40 μg of protein was prepared, loaded, and separated on 8, 10, 12 or 15% SDS-PAGE gels (depending on the molecular weight of the protein of interest) and then transferred onto a PVDF membrane (Thermo Scientific Pierce, Rockford, IL) using electrophoresis. Membranes were blocked for 1 hr in 5% non-fat dry milk and then incubated with total and phosphorylated p38, ERK 1/2, and SAPK/JNK antibodies (Cell Signaling, Boston, MA). Membranes were then washed and probed with HRP-Conjugated secondary antibodies (Jackson ImmunoResearch Laboratories, West Grove, PA). HRP activity was detected using an enhanced chemiluminescence (ECL) kit according to the manufacturer’s instructions (Thermo Scientific Pierce, Rockford, IL).

### Statistical Analysis

All experiments were performed at least in three different experiments. Statistical significance was analyzed using a paired student t-test and a 1-way analysis of variance (ANOVA) where applicable. Following ANOVA, the tukey-post hoc test was used to determine statistical significance between groups. A p- value less than 0.05 was considered to be statistically significant.

## Results

### Resolvins inhibit IL-1β secretion in THP1 cells in a dose-dependent manner

Macrophages are the major players involved in the initiation of the proinflammatory cytokine cascade. Macrophage mediated secretion of IL-1β, serves as an initiator of the proinflammatory response and results in upregulation of proinflammatory genes on IL-1β target cells [3]. Previously we have demonstrated that oxidant exposure leads to macrophage IL-1β secretion, ultimately resulting in enhanced alveolar epithelial cell permeability [6].

**Fig 1:**
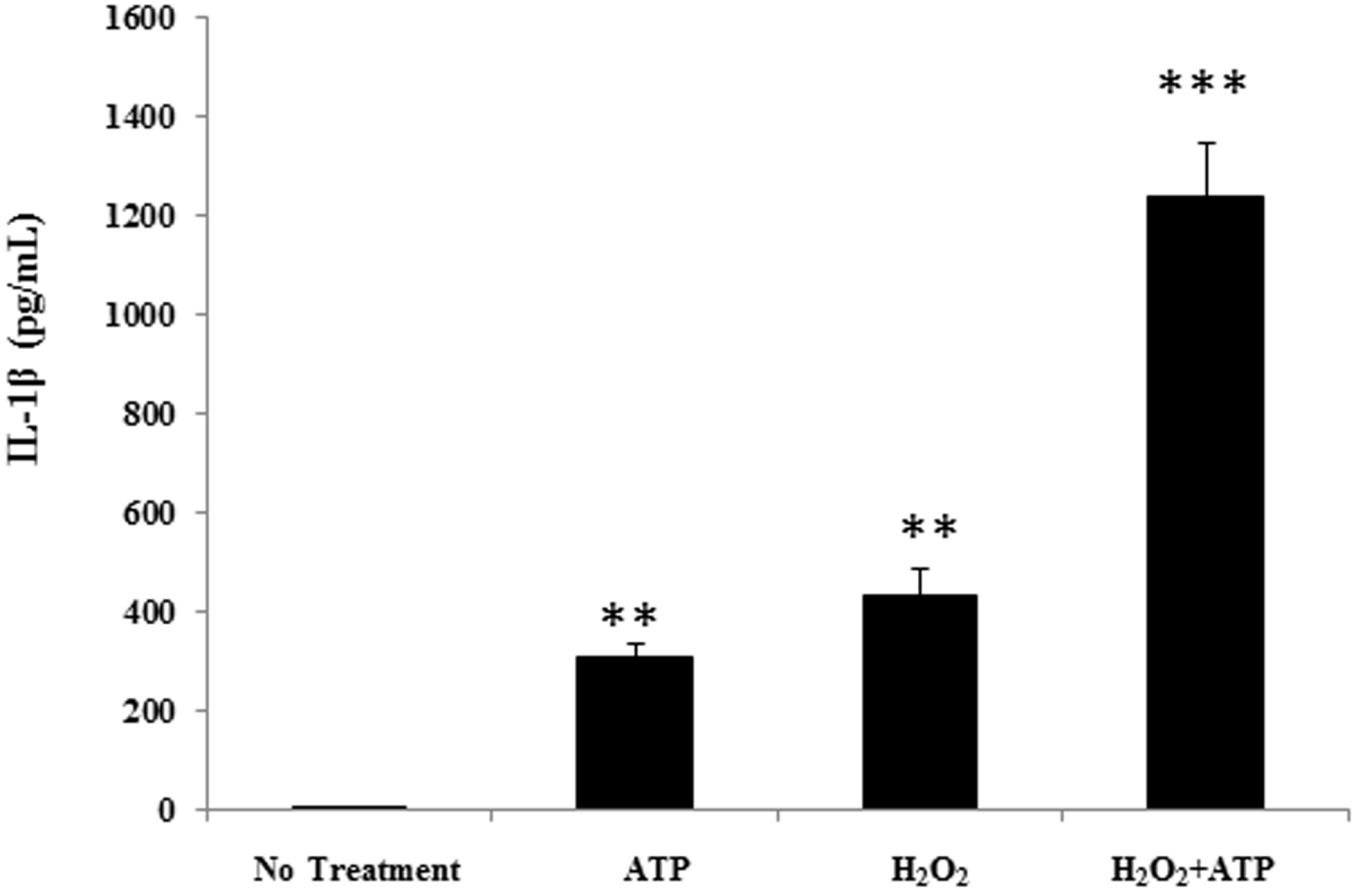
H_2_O_2_ and ATP Treatment Results in Enhanced IL-1β Secretion in THP-1 Macrophages. In order to reconstruct a physiologically relevant model of H_2_O_2_ induced macrophage secretion, primed THP-1 macrophages were treated with ATP (5 μM), H_2_O_2_ (10 mM) or combination of both ATP and THP1 for 1 hr. IL-1β released into the cell culture supernatant was analyzed. One way ANOVA was used with a tukey post-hoc test, where a p-value < 0.05 was deemed statistically significant (n = 4). Significance: ** = p < 0.01, *** = p < 0.001, vs no treatment control.

**Fig 2:**
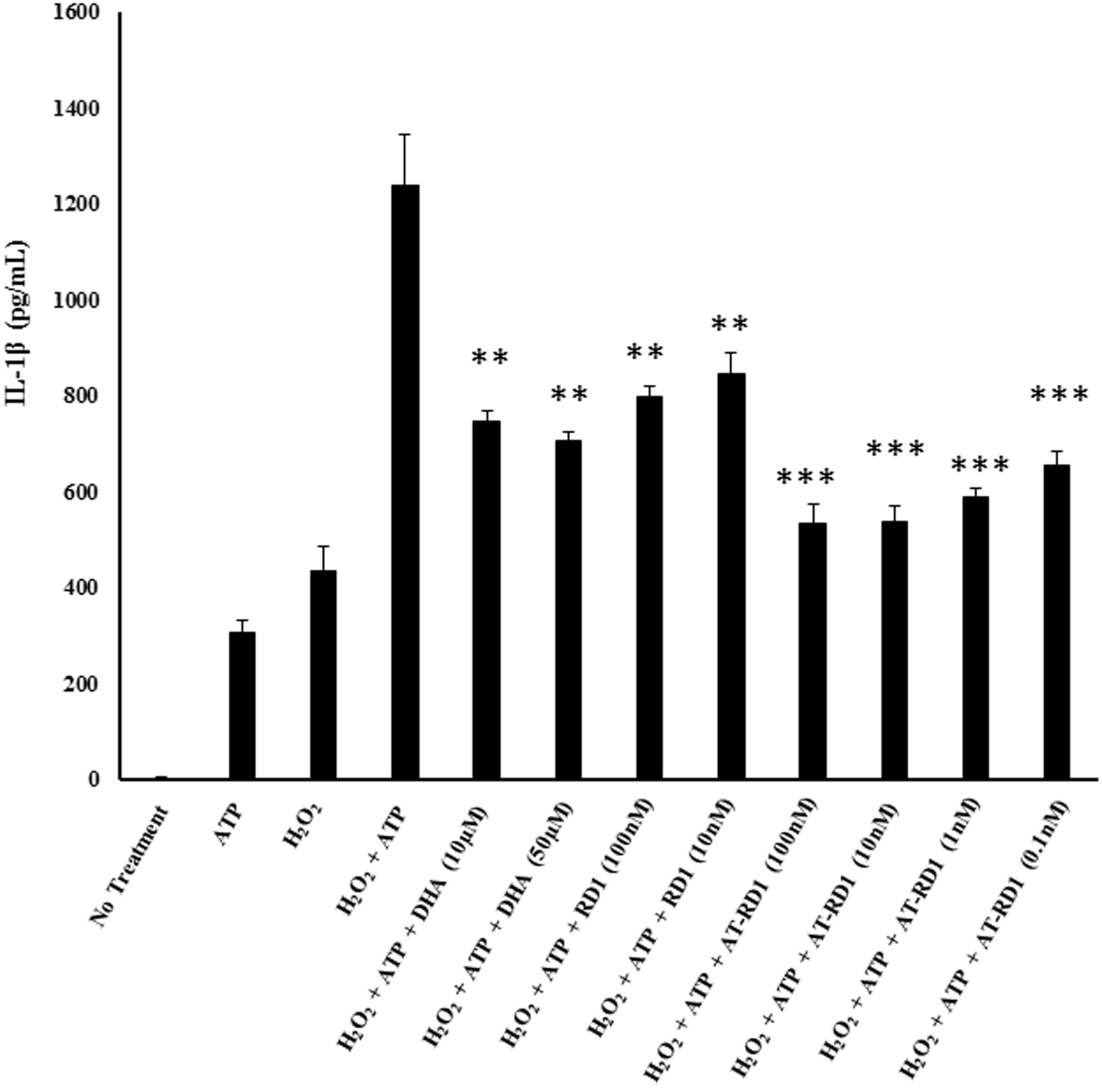
Omega-3 Treatment results in decreased H_2_O_2_-induced IL-1β secretion in THP1 macrophages. In order to assess the therapeutic effects of omega-3 fatty acids DHA and EPA as well as resolvin metabolites on IL-1β secretion, primed THP-1 macrophages were treated with ATP (5 μM), H_2_O_2_ (10 mM) or combination of both ATP in the presence or absence of EPA, DHA, RvD1 and AT-RvD1 at indicated doses for 1 hr. IL-1β release into the cell culture supernatant was analyzed. One way ANOVA was used with a tukey post-hoc test, where a p-value < 0.05 was deemed statistically significant (n = 4). Significance: ** = p < 0.01, *** = p < 0.001, vs no treatment control.

In order to see whether resolvins can attenuate oxidant mediated IL-1β secretion in macrophages, THP-1 monocytes were differentiated into macrophages and treated with hydrogen peroxide and ATP, both well-known inducers of IL-1β secretion in macrophages (Fig 1). We felt that this model of oxidant stress was physiologically relevant because our previous reports have shown that hyperoxia signaling leads to enhanced extracellular ATP release which serves as an adjuvant to oxidative stress to further enhance cytokine secretion [6]. Treatment with resolvin precursors DHA and EPA resulted in a significant reduction of resolvin in IL-1β secretion following oxidant-ATP treatment. Resolvins also leads to a significant decrease in IL-1β secretion with AT-RvD1 having the most significant decrease. More importantly these resolvins were able to accomplish significant attenuation of IL-1β secretion at the low nanomolar ranges in comparison to their parent fatty acids which achieved significance at the micromolar dosage (Fig 2).

**Fig 3:**
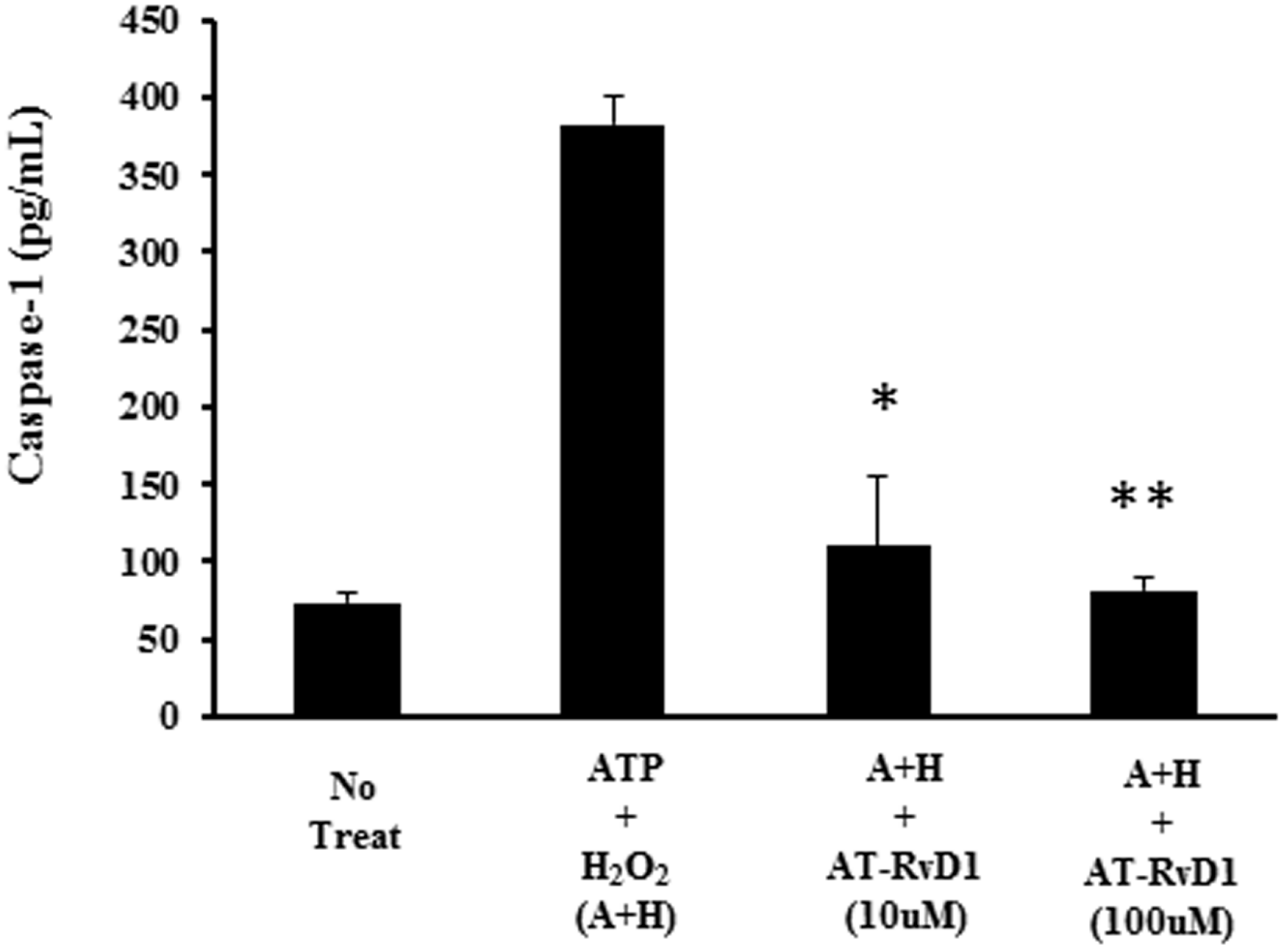
Resolvin Treatment results in decreased H_2_O_2_-induced caspase-1 activation in THP1 macrophages. In order to assess the role of RvD1 and AT-RvD1 on IL-1β processing enzyme caspase-1, primed THP-1 macrophages were treated with ATP (5 μM), H_2_O_2_ (10 mM) or combination of both ATP in the presence or absence of RvD1 and AT-RvD1 at indicated doses for 1 hr. Activated caspase-1 into the cell culture supernatant was analyzed by ELISA. One way ANOVA was used with a tukey post-hoc test, where a p-value < 0.05 was deemed statistically significant (n = 3).

To further confirm this we looked at resolvins ability to inhibit the activity of caspase-1, the primary processing enzyme. Results showed that resolvins RvD1 and AT-RvD1 show significant inhibition of caspase-1 activity following oxidant-ATP mediated activation (Fig 3). Taken together, these results highlight an important role for resolvins in decreasing the release of early macrophage mediated cytokine IL-1β in oxidant related injury.

### Resolvin treatment results in decreased macrophage induced alveolar epithelial cytokine secretion

**Fig 4:**
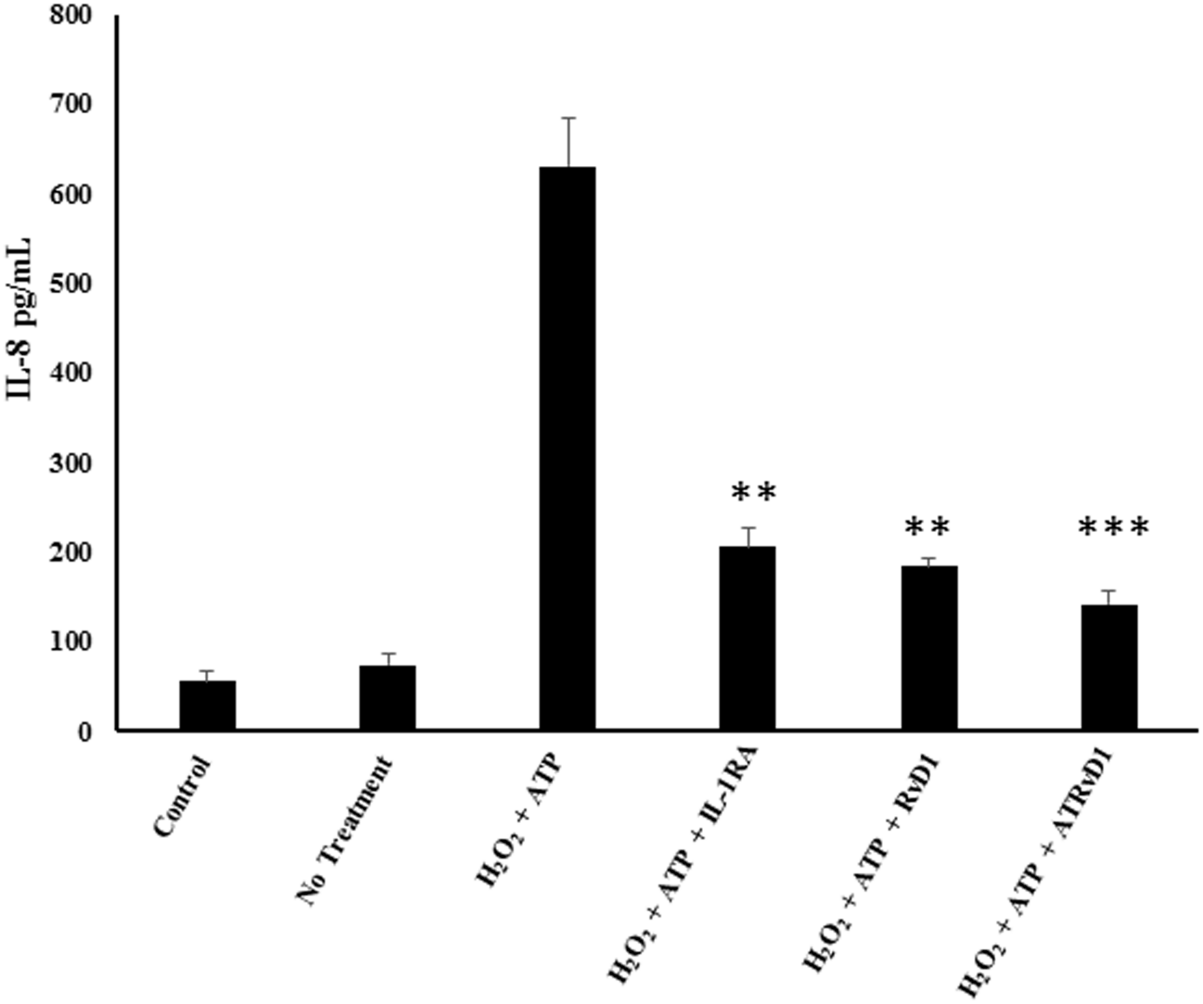
Resolvin Treatment leads to reduced macrophage induced alveolar epithelial cytokine secretion. THP-1 macrophages were treated with ATP + H_2_O_2_ in the presence or absence of RvD1 (100 nM) and AT-RvD1 (100 nM). Following treatment, supernatant from each macrophage group was collected and used to treat A549 cells for 6 hours. After treatment, supernatant from alveolar epithelial cells was collected and analyzed for interleukin-8 via ELISA. One way ANOVA was used with a tukey post-hoc test, where a p-value < 0.05 was deemed statistically significant (n = 4). Significance: ** = p < 0.01, *** = p < 0.001, vs no treatment control.

Our previous reports have demonstrated that oxidant mediated macrophage cytokine secretion results in increased permeability of alveolar epithelial cells. Previous reports have also highlighted that this interaction between the macrophages and epithelial cells are cytokine dependent. Since polarization of epithelial cells leads to an enhanced inflammatory state and is an important part of the inflammatory cascade, we wanted to assess resolvins’ ability to blunt the macrophage to epithelial cell interaction. We also wanted to assess whether the protective role was mediated through reduced IL-1β. To test this THP-1 monocytes were differentiated and treated with ATP and hydrogen peroxide in the presence or absence of resolvins RvD1 and AT-RvD1. Supernatants were collected and used to treat A549 cells for 1 hour. Following treatment supernatant was collected and analyzed for proinflammatory mediator and neutrophil chemoattractant IL-8. Results reveal that both RvD1 and AT-RvD1 blunted the ability of supernatant from oxidant-ATP treated cells to induce A549 cells to secrete IL-8 (Fig 4). Since AT-RvD1 showed a more significant increase in macrophage studies and is more resistant to enzymatic cleavage, we chose to use it as our primary molecule for resolvin investigation going forward in this dissertation.

### AT-RvD1 decreases IL-1β-mediated proinflammatory cytokine release in alveolar epithelial cells

**Fig 5:**
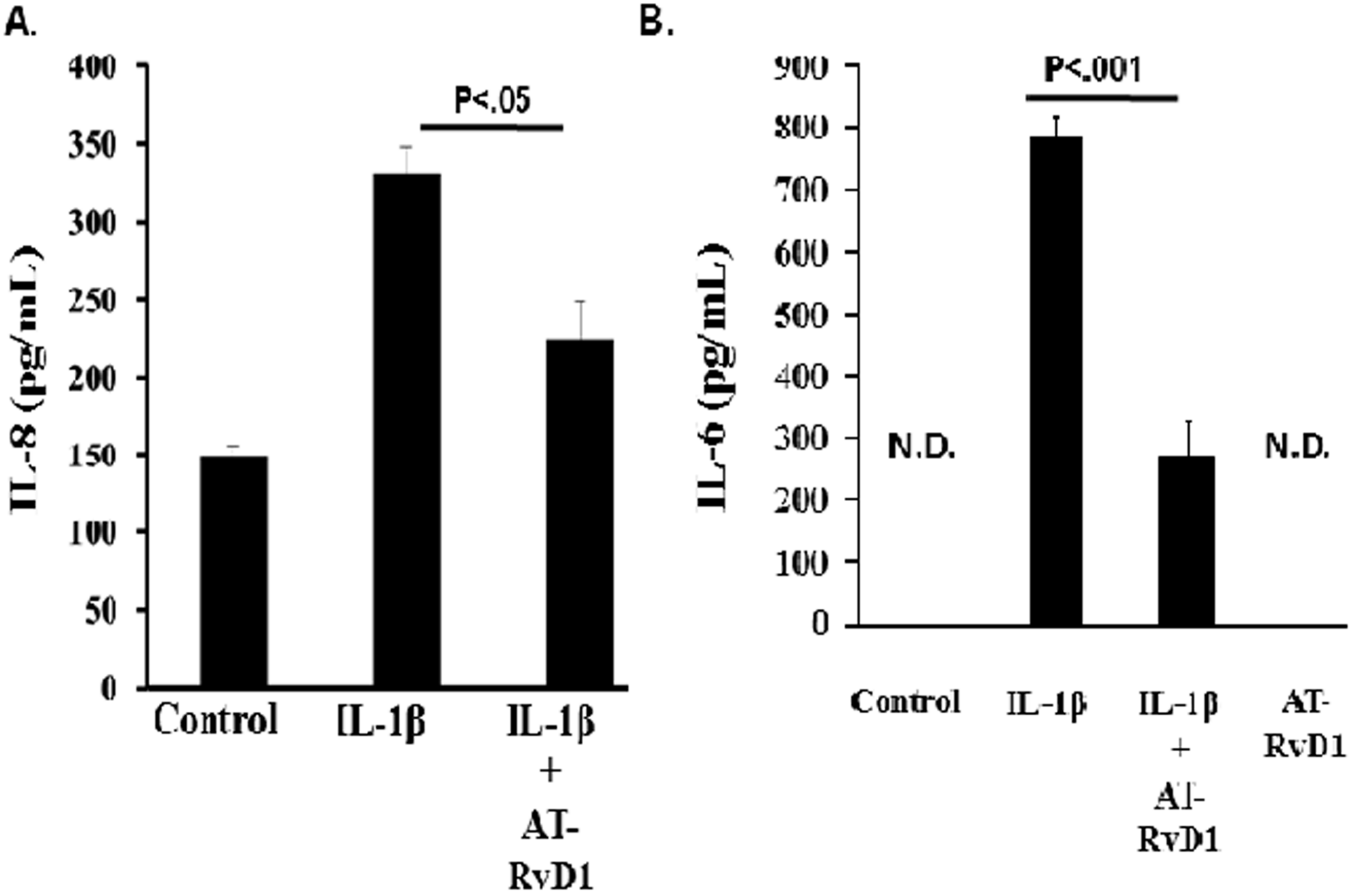
Aspirin-Triggered Resolvin D1 Attenuated IL-1β-induced Cytokine/Chemokine Secretion. A549 cells were seeded at a density of 0.5 x 10^6^ million cells per well in 12 well plates. When cells reached confluence, they were then serum starved and treated with IL-1β (10 ng/mL) in the presence or absence of aspirin-triggered resolvin D1 (AT-RD1, 100 nM) for 6 hours. Following treatment, cell culture supernatants were collected and the presence of (A) IL-8 and (B) IL-6 levels were analyzed by ELISA. A student t-test was used to determine statistical significance with p < 0.05 being statistically significant. ND = Not detected, limit of detection 2 pg/mL. n = 4 for both experiments.

Alveolar epithelial cells are activated when in the presence of proinflammatory molecules such as IL-1β [3]. Following activation, secretion of proinflammatory mediators such as IL-8 and IL-6 serves to propagate the inflammatory signal to the lung interstitium and vasculature [6, 32]. To determine whether AT-RvD1 inhibited the rate of proinflammatory cytokine secretion by alveolar epithelial cells, A549 cells were grown and the secretion of IL-6 and IL-8 were investigated. As shown in Figs 5A and 1B, A549 cells treated with IL-1β (10 ng/mL) display an increase in IL-6 and IL-8 secretion. However, this increase is attenuated for both cytokines in the presence of AT-RvD1 (100 nM). No significant difference was observed between the resolvin and control groups. While the release of both cytokines were significantly dampened with resolvin treatment, resolvins were able to reduce the IL-1β-mediated IL-6 (Fig 5B) by greater than 60% (p < 0.001) in comparison to IL-1β treatment alone.

### AT-RvD1 attenuates IL-1β-induced adhesion molecule expression in alveolar epithelial cells

**Fig 6:**
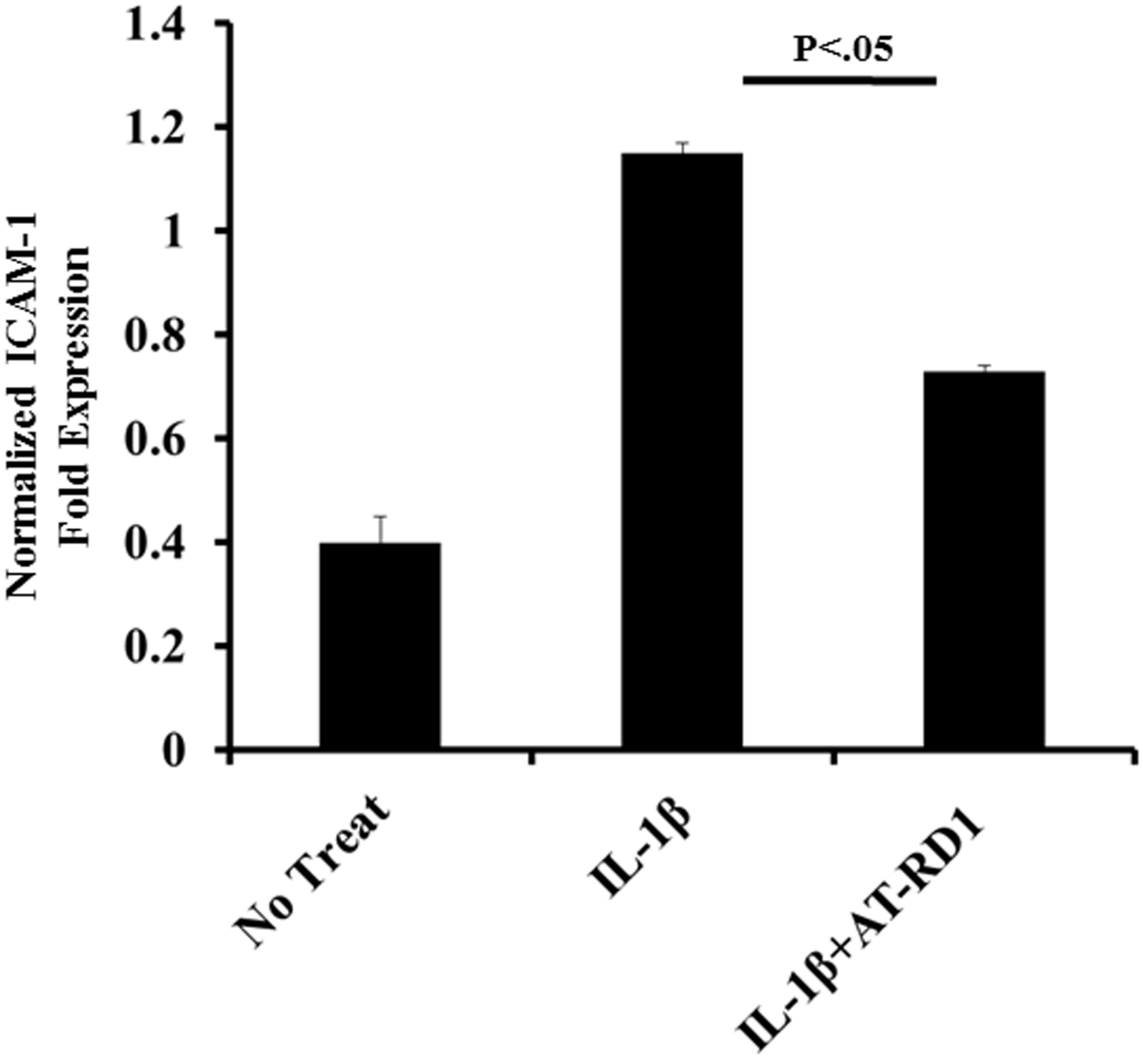
Aspirin-Triggered Resolvin D1 Attenuate IL-1β-induced Intercellular Adhesion Molecule-1 Expression. A549 cells were seeded at a density of 0.5 x 10^6^ million cells per well in 12 well plates. When cells reached confluence, they were then serum starved and treated with IL-1β (10 ng/mL) in the presence or absence of aspirin-triggered resolvin D1 (AT-RD1, 100 nM) for 6 hours. Following treatment, cells were lysed in Trizol and mRNA was extracted. Complimentary cDNA was produced and qPCR analysis was performed utilizing primers specific for ICAM-1. Statistical significance was determined using a standard student t-test with p < 0.05 being statistically significant (n = 5).

Proinflammatory cytokines such as IL-1β and IL-6 have been demonstrated to induce the expression of adhesion molecules such as intercellular adhesion molecule-1 (ICAM-1, CD54) [33, 34]. ICAM-1 is a transmembrane protein that plays an important role in the transepithelial migration of leukocytes into the epithelium. We hypothesized that the reduced cytokine secretion would result in decreased ICAM-1 expression. To investigate the effect of resolvins on IL-1β-induced A549 ICAM-1 expression, A549 cells were treated with IL-1β (10 ng) in the presence or absence of aspirin-treated resolvin D1 (100 nM). Following IL-1β treatment, ICAM-1 expression increased to its maximal value. 10 minute pretreatment of A549 cells with AT-RvD1 prior to IL-1β led to a 35% decrease (p < 0.05) in ICAM-1 expression (Fig 6).

### AT-RvD1 inhibits IL-1β-mediated monocyte and alveolar epithelial cell adhesion

**Fig 7:**
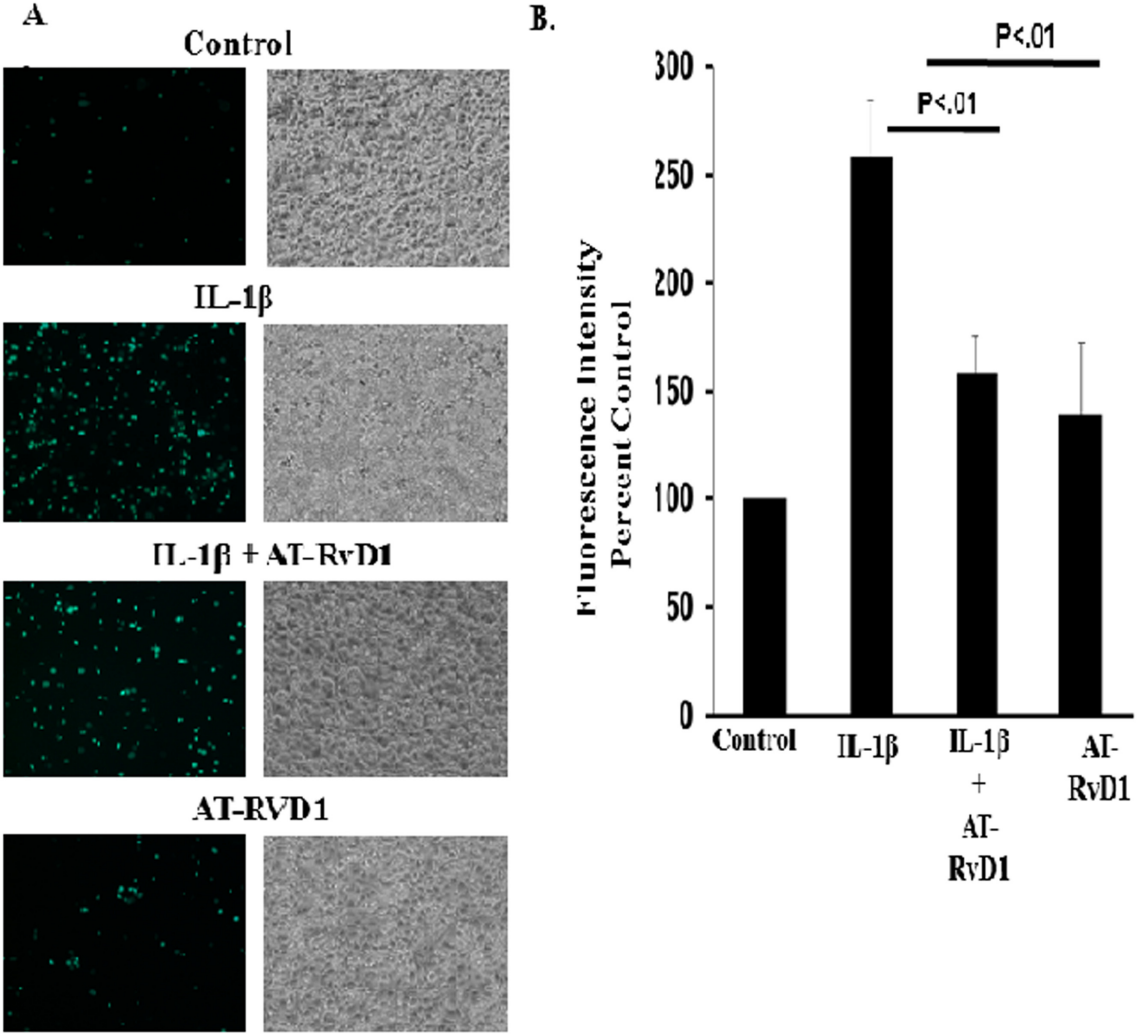
Aspirin-Triggered Resolvin D1 Attenuated IL-1β-mediated leukocyte-epithelial cell adhesion. A549 cells were seeded in A) 12 well at a density of 0.75 x 10^6^ or B) 96 well plates at a density of 0.1 x 10^6^ cells per well. Following serum starvation the cells were treated with IL-1β (10 ng/mL) in the presence or absence of aspirin-triggered Resolvin D1 (100 nM) for 6 hours. Following IL-1β treatment, calcein AM loaded THP1 cells were co-incubated with the alveolar epithelial cells for 30 minutes. Unbound attached monocytes were washed away with media and leukocyte attachment was observed via (A) fluorescent microscopy and (B) quantified with a reading of mean fluorescence intensity at Ex: 495 Em: 515. Statistical significance was measured with a one way ANOVA with a tukey post-hoc test, where p < 0.05 was designated as statistically significant (n = 3).

Circulating leukocytes extravasate out of vasculature and migrate into damaged tissue following acute injury. This is aided by the secretion of proinflammatory molecules such as IL-1β, IL-8, and IL-6 [35]. Once out of the vasculature, adhesions molecules such as ICAM-1 play a key role in the extravasation into the injured lung tissue [36]. Since resolvins decreased cytokine and ICAM-1 secretion and expression, respectively; we hypothesized that resolvins would decrease leukocyte adhesion to alveolar epithelial cells. To investigate this, A549 cells were treated with IL-1β (10 ng/mL) in the presence or absence of AT-RvD1 (100 nM). Following IL-1β treatment, calcein AM loaded THP1 monocytes were loaded into the wells containing A549 cells and co-cultured for 30 minutes. Image analysis shows that monocyte adhesion (as seen with green fluorescent dots) was significantly enhanced with IL-1β treatment alone in comparison to control. Monocyte adhesion was significantly diminished when A549 cells was pretreated with AT-RvD1 (Fig 7A). Treatment with AT-RvD1 alone did not significantly enhance monocyte adhesion. In a separate experiment, the adhesion assay was reproduced in 96 well plates and relative fluorescent intensity was measured to further quantify our results. Resolvin treatment resulted in greater than 50% reduction in IL-1β-mediated THP1-A549 cell adhesion in comparison to cells that were treated with IL-1β alone (Fig 7B). This further confirmed the difference seen in the fluorescence microscopy analysis. In an attempt to mitigate the effect of cellular variability, only confluent monolayer following IL-1β treatment was used for the adhesion assay. These monolayers are shown in the bright field images (Fig 7A). We did not detect a difference in monolayer consistency among the groups following IL-1β treatment.

### AT-RvD1 fails to rescue barrier dysfunction in alveolar epithelial cells

**Fig 8:**
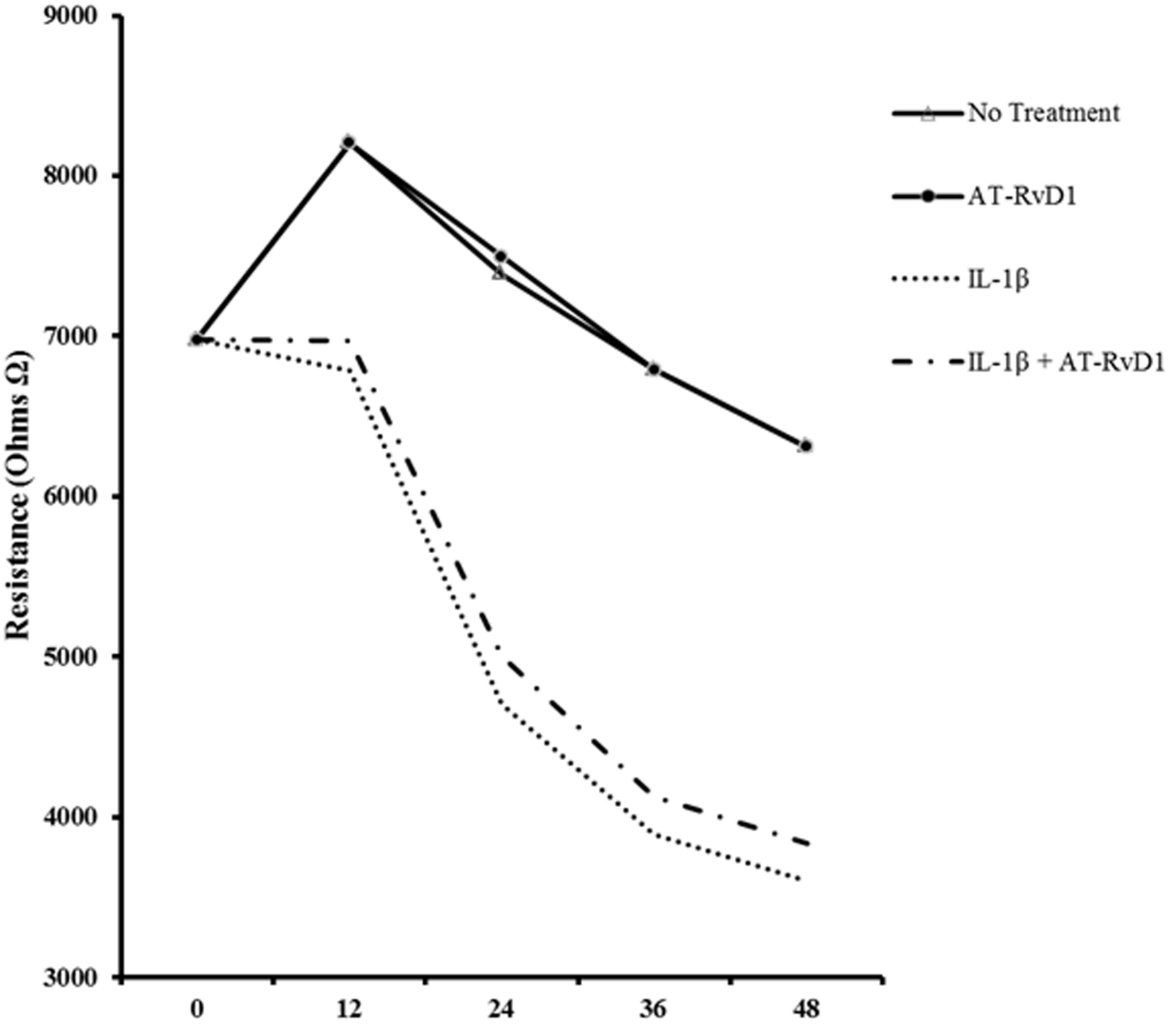
Aspirin-Triggered Resolvin D1 does not rescue barrier function in IL-1β-treated A549 cells. A549 cells were seeded in 8 well 8W1E ECIS arrays at a density of 5 x 10^4^ cells/mL. When cells reached confluence, they were treated with IL-1β (10 ng/mL) in the presence or absence of AT-RD1 (100 nM). Barrier resistance was recorded for 48 hours using ECIS. To eliminate variability; samples were treated, duplicated, and recorded 4 times at each time point. Statistical significance was measured with a one way ANOVA with a tukey post-hoc test, where p < 0.05 was designated as statistically significant (n = 4).

Alveolar epithelial cells form alveolar capillary barrier between the alveolar epithelium and the lung microvasculature. This barrier function of the alveolar epithelium also preserves the microenvironment of the alveoli and alveolar capillaries. Disruption of this barrier in the presence of proinflammatory mediators such as IL-1β leads to alveolar thickening, pulmonary edema, and diminished gas exchange [3, 7, 15, 37–39]. To assess the effect of resolvins on IL-1β-induced barrier dysfunction, transepithelial permeability (TER) was analyzed using electric cell-substrate impedance sensing (ECIS). A549, seeded in ECIS electrode arrays, were treated with IL-1β (10 ng/mL) in the presence or absence of aspirin-triggered resolvin D1 (100 nM) (Fig 8). TER was recorded by the ECIS system for 48 hours. Both resolvin alone and control treatment showed a small decrease in TER after 48 hours. There was no significant difference in TER between the groups treated with IL-1β and resolvins in comparison to those treated with IL-1β alone. Resolvin pretreatment led to a marginal increase in barrier resistance caused by IL-1β in comparison with IL-1β treatment alone.

### AT-RvD1 attenuates IL-1β-induced MAP Kinase Phosphorylation

**Fig 9:**
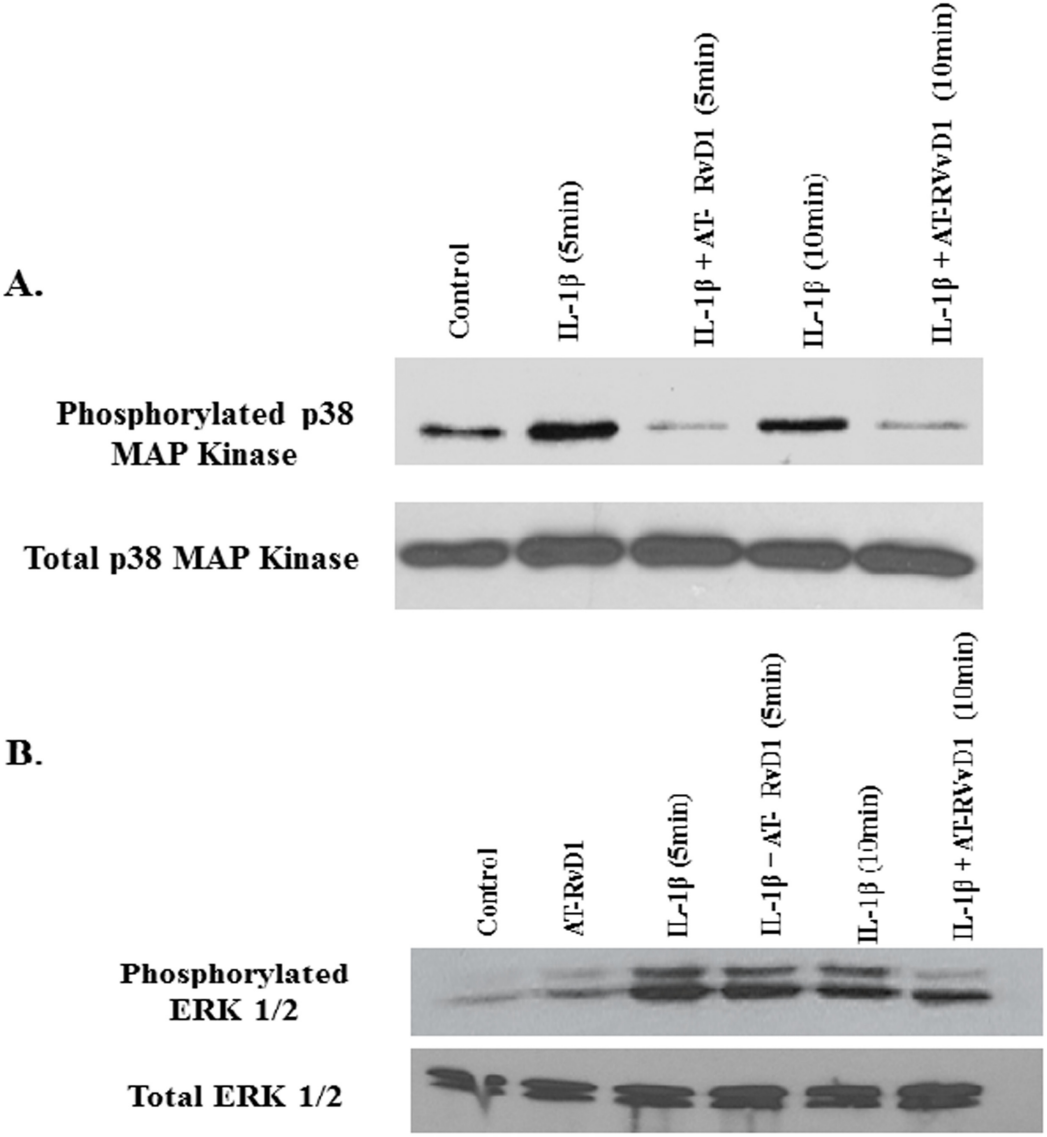
Resolvins regulate IL-1β-induced p38 and ERK 1/2 activity in alveolar epithelial cells. A549 cells were seeded at a density of 0.5 x 10^6^ million cells per well in 12 well plates. When cells reached confluence, they were then serum starved and treated with IL-1β (10 ng/mL) in the presence or absence of aspirin-triggered resolvin D1 (AT-RvD1, 100 nM) for 6 hours. Following treatment, cells were harvested and lysed with RIPA buffer. Western blot analysis was performed and the samples were probed for the presence of total and phosphorylated (A) p38 and (B) ERK 1/2 MAPKs using enhanced chemiluminescence. n = 3 for both set of experiments.

Mitogen activated protein kinases (MAPKs) are important regulators of the immune response in many myeloid and non-myeloid lineages such as alveolar epithelial cells. When activated these proteins play an important role in activating transcription factors that are necessary for transcription of proinflammatory events following injury such as cytokine production, barrier dysfunction, and adhesion molecule expression. The binding of IL-1β to its receptor, (IL-1R), leads to the activation of various pathways including those of MAPKs. Since we observed a change in IL-6 and IL-8 secretion as well as a decrease in ICAM-1 expression, we hypothesized that resolvins decrease the inflammatory effects of alveolar epithelial cells through decreased MAPK activation. To test this, A549 cells were treated with IL-1β (10 ng/mL) in the presence or absence of aspirin-triggered resolvin D1 (100 nM). Following treatments, cell lysates were collected for Western blot analysis of p38, ERK 1/2, and SAPK/JNK MAPK. These three MAPKs play a key role in propagating the inflammatory signal following IL-1β stimulation. AT-RvD1 pretreatment decreased p38 and to a lesser extent ERK phosphorylation at 5 and 10 minutes (Fig 9A and 9B). No change was observed between the control and AT-RvD1 alone samples. AT-RvD1 pretreatment failed to inhibit the phosphorylation of SAPK/JNK following IL-1β treatment at both 5 and 10 minutes. Taken together, these results suggest that resolvins modulate alveolar epithelial activation through a decrease in p38 and ERK 1/2 MAPKs.

## Discussion

IL-1β is known to be one of the earliest and most bioactive cytokines secreted following acute injury [3]. Release of this potent proinflammatory molecule causes up regulation of proinflammatory pathways on epithelial and endothelial cells leading to cytokine secretion [6, 35, 40]. This cytokine secretion is key to propagating the inflammatory signal beyond the airways and into the vasculature. We therefore investigated the effect of resolvins to curb inflammatory cytokine IL-6 and chemokine IL-8/CXCL8. These mediators, through paracrine, and autocrine signaling play a key role in persistence of the inflammatory phenotype [41–45]. We demonstrated the ability of resolvins to significantly curb the secretion of these proinflammatory cytokines. Further, we show that this decrease in cytokines by AT-RvD1 also correlated with a decrease in the expression of intercellular adhesion molecule-1 (ICAM-1). Once secreted into the extracellular milieu these inflammatory cytokines serve to upregulate important adhesion molecules that are used by migrating leukocytes to enter into the injured tissue and further prolong the inflammatory cycle [46]. These leukocytes such as neutrophils and monocytes are rich in proinflammatory mediators and add to the multifunctional inflammatory sequence that is not easily overcome naturally in inflammatory lung diseases. Our results tether AT-RvD1 to reduced proinflammatory molecules; however, we did not know the functional consequences of this reduced pro-inflammatory profile.

Since administration of AT-RvD1 significantly decreased the factors that play a role in monocyte recruitment such as adhesion molecule expression and cytokine secretion, we wanted to investigate IL-1β-induced alveolar epithelial cell-leukocyte interaction through adhesion. Monocytes and neutrophils serve as key first responders to the site of injury and can also be implicated as culprits in the overactive immune response [36, 47]. Inflammatory monocytes in the presence of inflammatory mediators such IL-1β, migrate into the tissue and differentiate into macrophages and ultimately foam cells in early injury [48]. These differentiated monocytes have a much longer lifespan than infiltrating neutrophils and serve to consistently reinvent the proinflammatory signal in uncontrolled inflammation seen in ALI [40]. Therefore, we wanted to investigate the effect of resolvins on the attachment to an IL-1β stimulated alveolar epithelium, citing the fact that IL-1β induces leukocyte attachment through adhesion molecule upregulation. In line with its effects on cytokine secretion and ICAM-1 expression, AT-RvD1 significantly diminished the effect of IL-1β to induce alveolar epithelial cell and monocyte adhesion. This correlates with previous reports that demonstrate resolvins as key mediators of leukocyte influx and barrier resistance [28, 29]. By our hands, however, we were not able to show an improvement in IL-1β-mediated alveolar epithelial barrier dysfunction with resolvin treatment.

MAP kinases are key molecules found in a variety of cells involved in the immune response. While different MAP kinases are important in different cells, the three predominant map kinases (p38, ERK, and SAPK/JNK) have all been shown to play a role in epithelial inflammatory signaling [33, 49]. AT-RvD1 treatment in accordance with previously reported effects of other resolvins on MAPKs showed a significant decrease in p38 and ERK phosphorylation and subsequent activation [29]. However, we did not observe a change in SAPK/JNK signaling. P38 and ERK have been shown to be responsible for the production of proinflammatory cytokines following IL-1β stimulation [50–52]. They have also been shown to be involved in adhesion molecule expression. Our results with resolvin pretreatment suggest a protective mechanism for resolvins through regulation of MAP kinase activity.

**Fig 10:**
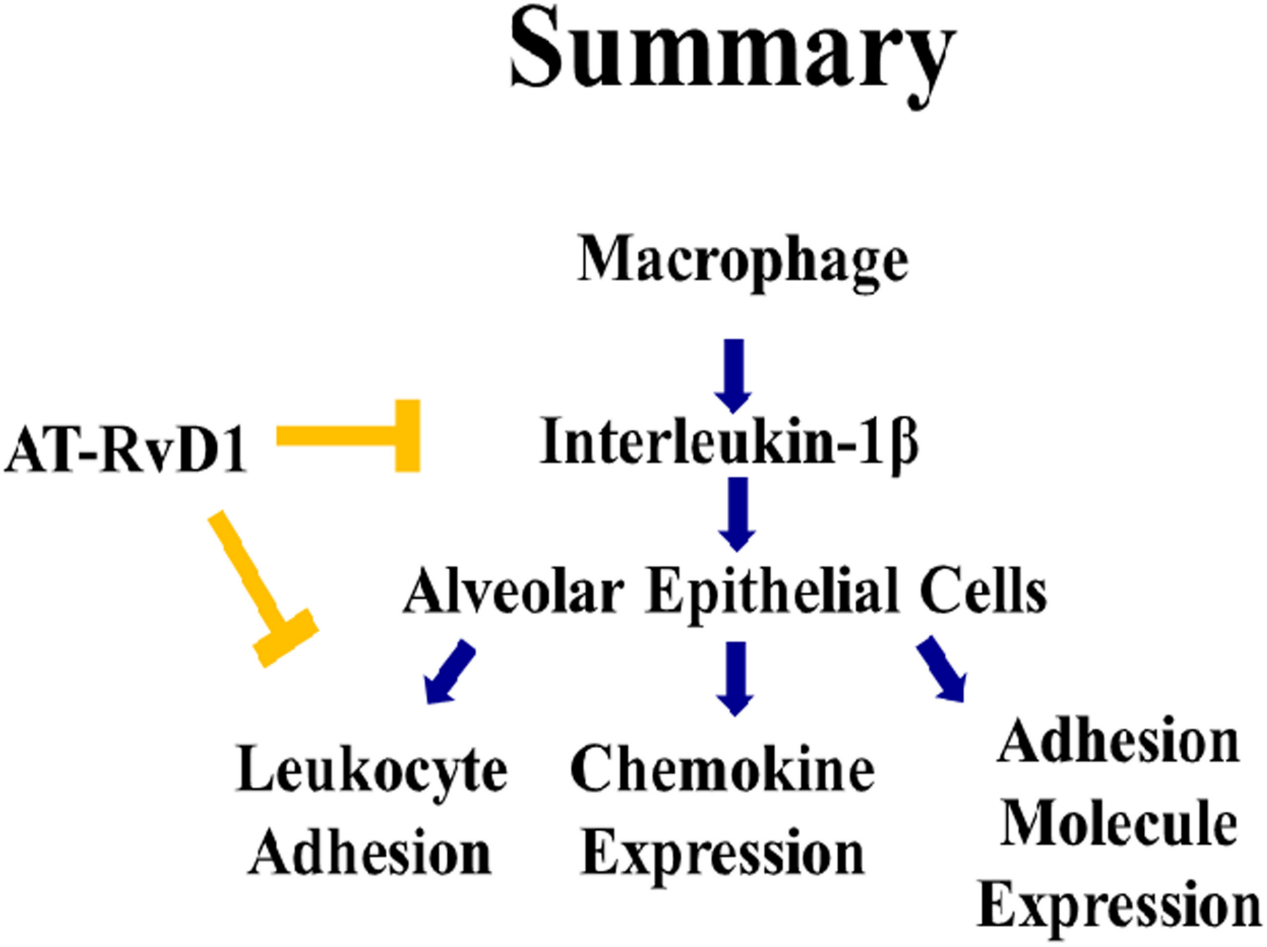
AT-RvD1 blunts macrophage and epithelial communication through reduced cytokine signaling. Exposure to oxidative stress leads to an enhanced secretion of proinflammatory cytokines, with IL-1β being the most bioactive for ALI patients. IL-1β secretion results in alveolar epithelial cell activation which is hallmarked by enhanced barrier function, cytokine secretion, and adhesion molecule expression. We found that AT-RvD1 was able to interrupt the macrophage to alveolar communication through the blockage of IL-1β signaling. Upon treatment of alveolar epithelial cells with IL-1β, there was an increase in inflammatory activation, which was significantly attenuated with AT-RvD1 treatment.

Inflammatory lung disorders such as cystic fibrosis or ALI feature significantly enhanced levels of proinflammatory cytokines as well as aggressive infiltration of leukocytes into the injured tissue [1, 40, 50, 53]. While alveolar macrophages play a key role in the secretion of early mediators such as IL-1β, the alveolar epithelium is an important source of inflammatory mediators as well and a key site of immune cell tissue interaction following injury [3]. Secretion and binding of IL-1β leads to barrier dysfunction, cytokine/chemokine secretion, and barrier dysfunction. Omega-3 (ω-3) free fatty acids have shown beneficial effects *in vitro* at curbing the immune response. Ω-3 derivatives termed ‘resolution-phase interaction products’ have also been demonstrated to curb the inflammatory phenotype seen in diseases such as COPD and aspiration pneumonia-induced ALI [27–29, 54]. In this article, we demonstrate for the first time the protective effects of aspirin-triggered resolvin D1 (AT-RvD1) in diminishing the inflammatory phenotype of these cells following IL-1β stimulation (Summary, Fig 10). Together, this report supports previous studies that show that resolvins are potent potential therapeutic for inflammation seen in ALI [24–26, 54–56]. More importantly, these experiments demonstrate a novel role of AT-RvD1 in the inhibition of IL-1β-induced alveolar epithelial cell activation.

## Supporting Information

S1 Fig AT-RvD1 Treatment does not lead to increased cytotoxicity in A549 cells. A549 cells were seeded at a density of 0.5 x 10^6^ million cells per well in 12 well plates. When cells reached confluence, they were treated with AT-RvD1 at the indicated doses for 24 hours. Following treatment cell viability was assessed using the trypan blue dye exclusion assay as previously described. Statistical significance was measured with a one way ANOVA with a tukey post-hoc test, where p < 0.05 was designated as statistically significant (n = 4).
(TIF)
