# Supplementary figures and images for: Resolvins Decrease Oxidative Stress Mediated Macrophage and Epithelial Cell Interaction through Decreased Cytokine Secretion
Source: PLoS One. 2015 Aug 28;10(8):e0136755. doi: 10.1371/journal.pone.0136755 (PMC4552682; doi:10.1371/journal.pone.0136755)

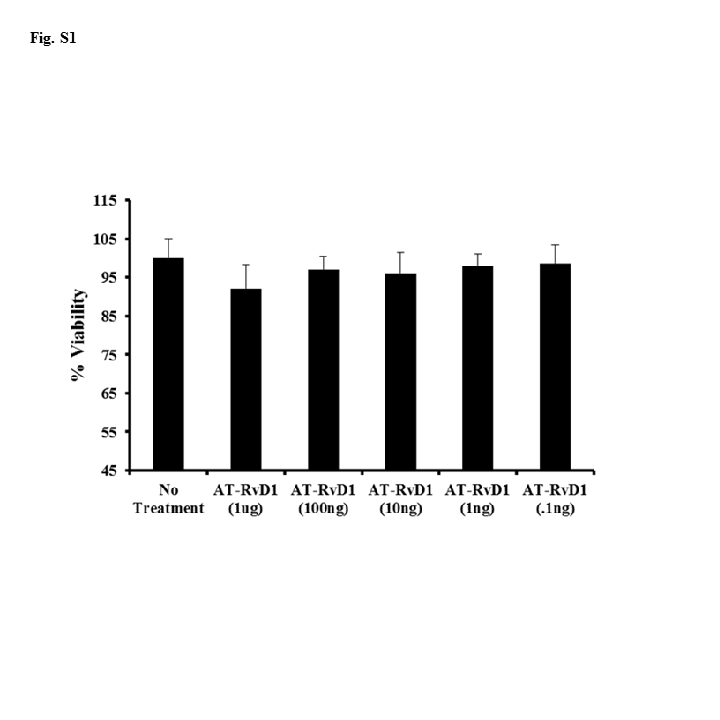

Supplement: S1 Fig — A549 cells were seeded at a density of 0.5 x 10^6^ million cells per well in 12 well plates. When cells reached confluence, they were treated with AT-RvD1 at the indicated doses for 24 hours. Following treatment cell viability was assessed using the trypan blue dye exclusion assay as previously described. Statistical significance was measured with a one way ANOVA with a tukey post-hoc test, where p < 0.05 was designated as statistically significant (n = 4). (TIF) [file pone.0136755.s001.tif]
